# Composition‐Controlled Cathode Protective Layer via Powder‐Atomic Layer Deposition for All‐Solid‐State Batteries

**DOI:** 10.1002/advs.202514583

**Published:** 2025-10-14

**Authors:** Kyu Moon Kwon, Dae Ho Kim, Ha Yeon Kwon, Joungwon Park, Kyoung Hwan Kim, Hwi‐Yeol Park, Hyo Rang Kang, Tae Joo Park

**Affiliations:** ^1^ Department of Materials Science and Chemical Engineering Hanyang University Ansan 15588 South Korea; ^2^ Battery Material TU, Samsung Advanced Institute of Technology (SAIT) Samsung Electronics Co., Ltd. Suwon 16678 South Korea; ^3^ Nanocamp Inc. Chungju 27315 South Korea; ^4^ ALPES Co., Ltd. Ansan 15588 South Korea

**Keywords:** all‐solid‐state batteries, atomic layer deposition, cathode protective layer, lithium zirconium oxide, powder ALD

## Abstract

Controlling the ionic and electronic conductivities of the protective layers on cathode active materials (CAMs) is critical for interface stabilization with sulfide‐based solid electrolytes in all‐solid‐state batteries (ASSBs). In this study, lithium zirconium oxide layers with varying compositions are grown on LiNi_0.8_Co_0.1_Mn_0.1_O_2_ using an O_3_‐based atomic layer deposition process. The ionic conductivity reaches 2.20 × 10^−7^ S cm^−1^ at 25 °C, and the electronic conductivity ranges from 10^−10^ to 10^−7^ S cm^−1^ depending on composition. These composition‐dependent transport properties directly affect electrochemical performance, leading to a 4.5% difference in initial Coulombic efficiency and a 37% difference in long‐term retention. Compared to their uncoated counterparts, the coated CAMs exhibited 6.7% and 43% increases in initial efficiency and capacity retention of the cells, respectively. This study establishes a quantitative correlation between the protective layer's composition and battery performance, emphasizing that composition control is a key strategy for interfacial engineering in sulfide‐based ASSBs.

## Graphical abstract

Composition control of the LiZrO protective layer via atomic layer deposition modulates its ionic and electronic conductivities, directly affecting interfacial stability and electrochemical performances of the sulfide‐based all‐solid‐state batteries. This study establishes the composition of the protective layer as an essential design axis for cathode interface engineering, beyond conventional parameters such as film thickness and coating method.

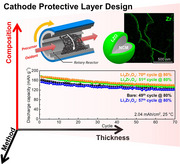

## 1 Introduction

Sulfide‐based solid electrolytes (SEs) have attracted significant attention for use in next‐generation energy storage systems because of their high ionic conductivities of up to 1 mS cm^−1^ and compatibility with cold sintering processes.^[^ ^1^, ^2^ ^]^ However, their commercialization is hindered by several technical challenges, among which interfacial side reactions between cathode active materials (CAMs; e.g., LiNi*_x_* Co*_y_* Mn*_z_* O_2_) and sulfide‐based SEs (e.g., Li_6_PS_5_Cl) are particularly critical. These interfacial reactions, driven by the mismatch in the electrochemical stability windows of the two materials, are a major cause of capacity fading and shortened cycle life in all‐solid‐state batteries (ASSBs).^[^ ^3^, ^4^, ^5^, ^6^, ^7^ ^]^ One widely studied approach to mitigate this issue is coating the CAM surface with a protective layer. Coating CAMs with a Li‐ion‐conducting material that is electrochemically stable with the SE can effectively suppress unwanted redox reactions.^[^ ^7^, ^8^, ^9^, ^10^, ^11^, ^12^ ^]^

For a protective coating to function effectively, it must fully cover the CAM surface to prevent direct contact with the SE.^[^ ^7^ ^]^ Protective layers have conventionally been grown using wet chemical processes, which face limitations in controlling the coating thickness and achieving uniform coverage of the CAM particles.^[^ ^13^, ^14^ ^]^ In contrast, atomic layer deposition (ALD) enables angstrom‐level control of film thickness owing to its self‐limiting reaction mechanism. Moreover, ALD provides uniform, conformal, and pinhole‐free thin films, even on complex or high‐aspect‐ratio structures, making it one of the most promising techniques for protective layer coatings.^[^ ^15^, ^16^, ^17^, ^18^, ^19^ ^]^ Recently, various Li metal oxides (LMOs) such as LiNbO_3_,^[^ ^20^ ^]^ Li_2_ZrO_3_,^[^ ^21^ ^]^ and LiAlZnO,^[^ ^22^ ^]^ have been employed as protective layers on CAMs using ALD, leading to significant improvements in the interfacial stability and cycle life of ASSBs. However, most of these studies focused on coating thickness optimization, and the impact of the protective layer's composition on the electrochemical properties of ASSBs remains relatively underexplored.

The composition of the protective layer significantly influences the ionic and electronic conductivities. Hu et al.^[^ ^23^ ^]^ reported that in amorphous Li*_x_* TaO*_y_* films grown by ALD, the ionic conductivity varied by up to two orders of magnitude, depending on the Li/Ta composition ratio (*x* value). The ionic conductivity increased as *x* increased from 0.32 to 0.98, but decreased under Li‐rich conditions (*x* = 1.73). Similarly, Jonderian et al.^[^ ^24^ ^]^ demonstrated that, in Li–B–Si–O glass electrolytes synthesized via a sol‐gel method, both ionic and electronic conductivities increased with higher Li concentration. These composition‐driven conductivity changes directly affect battery performance. The ionic conductivity of the protective layer affects Li^+^ transport kinetics at the interface, which directly impacts cell performance metrics, including discharge capacity, rate capability, and cycle life.^[^ ^25^ ^]^ Meanwhile, higher electronic conductivity tends to allow more redox reactions at the interface, resulting in a loss of reversible capacity.^[^ ^26^ ^]^ Conversely, conformal coating of the CAM surface with an insulating protective layer such as Al_2_O_3_ with extremely low electronic conductivity (≈10^−16^ S cm^−1^),^[^ ^27^ ^]^ can electrically isolate the cathode, hindering electron transport during charge–discharge processes.^[^ ^7^, ^28^ ^]^ Therefore, to improve the interfacial stability between the CAM and SE, as well as overall cell performance, it is essential to control the composition of the protective layer to tune both ionic and electronic conductivity.

In this study, the effect of the protective layer's composition on ionic and electronic conductivities was examined, and its influence on ASSB performances was demonstrated experimentally. To enable composition control, the growth behaviors of lithium zirconium oxide (LZO) film using different oxygen sources were compared, and three LZO protective layers with various compositions were fabricated via an ALD process with O_3_. The ionic and electronic conductivities of the layers were analyzed, and their impacts on the cell performances, including the initial discharge capacity, Coulombic efficiency (CE), rate capability, and cycle life, were evaluated.

## 2 Results and Discussion

**Figure 1:**
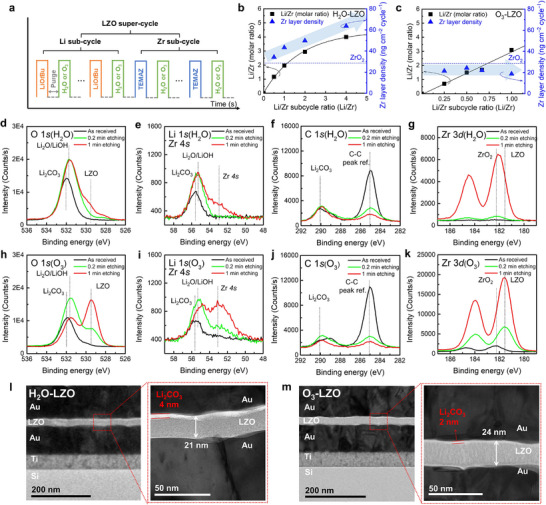
a) Schematic of the ALD supercycle method for LZO film deposition with varying Li/Zr subcycle ratios and different oxygen sources (H_2_O or O_3_). Composition change and deposition behavior of b) H_2_O‐LZO and c) O_3_‐LZO, expressed as Li/Zr molar ratio and Zr layer density per cycle as a function of Li/Zr subcycle ratio. The Zr layer density per cycle of ZrO_2_ is indicated by a blue dashed line. d,h) O 1*s*, e,i) Li 1*s* with Zr 4*s*, f,j) C 1*s*, and g,k) Zr 3*d* core‐level spectra of LZO films (Li/Zr molar ratio = 2:1) deposited using H_2_O and O_3_ as the oxygen sources. Cross‐sectional HRTEM images of l) H_2_O‐LZO and m) O_3_‐LZO, showing total thickness and surface Li_2_CO_3_ layer.

**Figure** **1**a shows a schematic of the supercycle method used for the deposition of the LZO films. In this method, ALD cycles for multiple materials are divided into subcycles, which are then combined in integer ratios to form supercycles, enabling precise control over the film composition. By adjusting the subcycle ratio, the film composition can be tuned, and the total number of supercycles determines the overall thickness of the film. Figure 1b,c show the growth behavior of the LZO films (H_2_O‐LZO and O_3_‐LZO, respectively) grown on Si substrates using H_2_O and O_3_ as the oxygen sources. The Li/Zr molar ratio (i.e., the LZO composition) was determined using inductively coupled plasma mass spectrometry (ICP‐MS), and the Zr layer density per total number of Zr subcycles (Zr layer density per cycle) was measured using energy‐dispersive X‐ray fluorescence (EDXRF). These values were plotted as a function of the Li/Zr subcycle ratio, which is defined as the total number of Li subcycles divided by the total number of Zr subcycles. All subsequent references to LZO compositions in this study are based on the ICP‐MS values. In the case of H_2_O‐LZO (Figure 1b), the Li/Zr molar ratio showed a saturating trend with increasing Li/Zr subcycle ratio. In addition, the Zr layer density per cycle was higher than that of pure ZrO_2_ (blue dashed line), and the layer density increased further with higher Li/Zr subcycle ratios. This behavior is attributed to a reservoir effect, in which H_2_O pulsed during the Li subcycle was physisorbed onto the hygroscopic LiOH surface and not completely removed during the purge step, thereby inducing extra reactions upon the subsequent pulsing of the Zr precursor.^[^ ^29^, ^30^ ^]^ Consequently, the Zr layer density per cycle exceeded that of pure ZrO_2_ films regardless of the subcycle ratio. As the Li/Zr subcycle ratio increased, more H_2_O was retained during the Li subcycle, leading to the increased formation of LiOH·H_2_O. The hydrates subsequently reacted with the Zr precursor, resulting in excess ZrO_2_ deposition, which saturated the Li/Zr molar ratio in the LZO films. These results indicate that when H_2_O is used as the oxygen source, effective control of the deposited film composition cannot be achieved by tuning the subcycle ratio. Moreover, such nonself‐limiting reactions degrade the uniformity and conformality of the LZO film. Residual H_2_O, if not completely purged, may induce structural and chemical degradation of the CAM.^[^ ^31^ ^]^ Therefore, ALD process with H_2_O is inappropriate for precise composition control and is also unsuitable for the deposition of protective layers on electrode surfaces, where a uniform and conformal coating is essential.

O_3_ is commonly used as an oxygen source in conjunction with H_2_O. Although previous studies employed Li 2,2,6,6‐tetramethyl‐3,5‐heptanedionate(Li(thd)) with O_3_ for the deposition of Li‐containing oxide films,^[^ ^32^ ^]^ its combination with Li *tert*‐butoxide (LiO*t*Bu) remains unexplored. Therefore, the growth behavior of LZO film was investigated using an O_3_‐based process, with LiO*t*Bu and O_3_ employed in the Li subcycle, and tetrakis(ethylmethylamino) Zr (TEMAZ) and O_3_ used in the Zr subcycle to eliminate any potential influence from H_2_O. The pulse times for both subcycles were optimized (Figure S1, Supporting Information). In the case of O_3_‐LZO (Figure 1c), unlike H_2_O‐LZO, which exhibited a saturation trend, the Li/Zr molar ratio increased linearly with increasing Li/Zr subcycle ratio. The Zr layer density per cycle showed minimal variation with the Li/Zr subcycle ratio and remained close to that of pure ZrO_2_ (blue dashed line). This result suggests that hydrate formation did not occur in the O_3_‐based process. Consequently, the Zr subcycle proceeded without additional reactions, and the ZrO_2_ deposition rate was independent of the Li subcycle. These findings confirm that the Li and Zr subcycles were decoupled, permitting the precise tuning of the film composition through subcycle ratio modulation under self‐limiting conditions. The linear relationship between the Li subcycle and LZO composition reflects the surface utilization model proposed by Nilsen et al.,^[^ ^33^ ^]^ highlighting the advantage of using O_3_ as an oxygen source for precise composition control in the ALD of LMOs.

Figure 1d–g,h–k show the various core‐level spectra of H_2_O‐LZO and O_3_‐LZO, respectively. Both samples were grown with the composition of Li_2_Zr_1_O_x_ to enable consistent comparison. The O 1*s* core‐level spectra of H_2_O‐LZO (Figure 1d) initially exhibited a Li_2_CO_3_ peak at the binding energy (BE) of 532 eV. Upon surface etching, a Li_2_O/LiOH peak emerged at the BE of 531.7 eV, while the Li_2_CO_3_ peak at the BE of 532 eV still remained. A similar trend was observed in the Li 1*s* core‐level spectra (Figure 1e), where the Li_2_CO_3_ peak at the BE of 55.7 eV was present both before and after 1 min of etching. The C 1*s* core‐level spectra (Figure 1f) also exhibited a Li_2_CO_3_ peak at the BE of 290 eV, and its intensity barely decreased after surface etching, suggesting that a thick Li_2_CO_3_ layer was formed at the surface, with Li_2_O/LiOH present beneath it. In the O 1*s* core‐level spectra, the characteristic LZO peak at the BE of 529.5 eV was hardly observed initially, but it appeared weakly after 1 min of etching. This trend was consistently observed in other core‐level spectra as well. In the Zr 4*s* core‐level spectra (Figure 1e), no peak was detected before 1 min of etching, after which a peak at the BE of 53 eV appeared. Similarly, in the Zr 3*d* core‐level spectra (Figure 1g), a weak ZrO_2_ peak at the BE of 182.2 eV emerged after 0.2 min of etching and became pronounced after 1 min. The absence of LZO peaks before surface etching, followed by their appearance afterward, indicates that this phase was obscured by surface layers of Li_2_CO_3_ and Li_2_O/LiOH. These surface species hindered the initial detection of the LZO phase despite its presence within the film. These results suggest that a homogeneous LZO phase was hardly formed. Instead, Li‐ and Zr‐containing phases existed separately, indicating surface‐level phase separation.

In contrast, the core‐level spectra of O_3_‐LZO (Figure 1h–k) exhibited different trends. In the O 1*s* core‐level spectra (Figure 1h), the Li_2_CO_3_ peak at the BE of 532 eV progressively diminished upon etching, while the Li_2_O/LiOH peak at the BE of 531.7 eV emerged after 0.2 min of etching and rapidly faded after 1 min. The Li_2_CO_3_ peak at the BE of 55.7 eV in the Li 1*s* core‐level spectra (Figure 1i) similarly showed a decrease in intensity upon surface etching. The Li_2_O/LiOH peak at the BE of 55.3 eV appeared simultaneously, but declined after 1 min. In the C 1*s* spectra (Figure 1j), the intensity of the Li_2_CO_3_ peak at the BE of 290 eV decreased steadily upon etching, suggesting that the surface carbonate layer was relatively thin compared to that of H_2_O‐LZO. The LZO peak at the BE of 529.5 eV in the O 1*s* core‐level spectra (Figure 1h) gradually intensified upon etching. A similar trend was observed in the peaks at the BE of 53 and 182.2 eV in the Zr 4*s* (Figure 1i) and Zr 3*d* (Figure 1k) core‐level spectra, respectively. Notably, in the Zr 3*d* core‐level spectra (Figure 1k), a ZrO_2_ peak (182.2 eV) was initially observed. However, after etching, strong LZO peaks emerged at 181.6 eV, indicating a shift toward a lower binding energy. This shift is attributed to the incorporation of Li─O bonds, which increased the electron density around the Zr ions,^[^ ^34^ ^]^ providing direct evidence for the formation of a homogeneous LZO phase.

Figure 1l,m present the cross‐sectional high‐resolution transmission electron microscopy (HRTEM) images of H_2_O‐LZO and O_3_‐LZO, respectively. In both samples, a surface layer that appeared brighter and with lower contrast was observed, corresponding to the Li_2_CO_3_ phase identified by XPS (Figures 1d–f,h–j). The thickness of this layer was ≈4 and 2 nm for H_2_O‐LZO and O_3_‐LZO, respectively. The facile formation of Li_2_CO_3_ layer in the ALD process with H_2_O has been reported previously,^[^ ^35^ ^]^ and similar behavior was observed in these analyses. Residual Li_2_CO_3_ phase on the surface or within the film may reduce the ionic conductivity,^[^ ^36^ ^]^ representing an additional drawback of the ALD process with H_2_O beyond the composition control limitations. Collectively, these results demonstrate that the ALD process with O_3_ offers enhanced chemical stability, as shown by both the reduced surface Li_2_CO_3_ layer thickness (≈50% vs H_2_O‐LZO) and the more uniform bonding states within the film. Moreover, the previously discussed linear relationship between the Li/Zr subcycle ratio and the resulting composition (Figure 1b,c) confirms that precise composition control can be achieved through subcycle modulation under self‐limiting reaction conditions. Additionally, both the H_2_O‐LZO and O_3_‐LZO films exhibited negligible carbon impurity within the bulk (Figure S2, Supporting Information), further confirming the suitability of the O_3_‐based process for the deposition of LZO protective layers compared with its H_2_O‐based counterpart.

**Figure 2:**
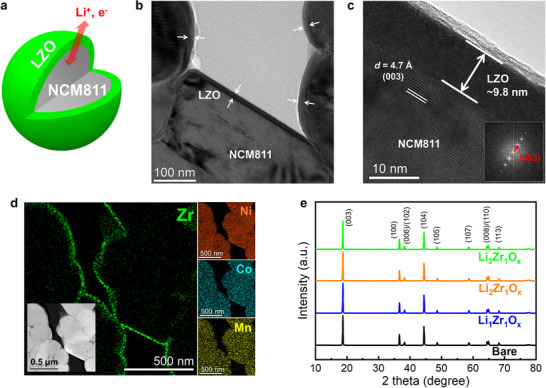
a) Core–shell structure of an NCM811 particle coated with an LZO protective layer via powder ALD. b,c) Cross‐sectional HRTEM images of the NCM811 particle with the LZO protective layer. An interlayer spacing of 4.7 Å is observed, consistent with the (003) plane of NCM811. The inset shows the FFT pattern obtained from the NCM811 region. d) HAADF‐STEM image (inset) and corresponding EDS elemental maps of Zr, Ni, Co, and Mn, confirming the conformal deposition of LZO protective layer on the surface of NCM811 particles. e) XRD patterns of NCM811 with and without various LZO protective layers.

**Figure** **2**a shows a schematic of LiNi_0.8_Co_0.1_Mn_0.1_O_2_ (NCM811) particle coated with an LZO protective layer via rotary‐type powder ALD with O_3_. The corresponding transmission electron microscopy (TEM) images of NCM811 particles with the LZO protective layer are shown in Figure 2b–d. Figure 2b,c show cross‐sectional HRTEM images that confirm the uniform LZO protective layer with a thickness of ≈9.8 nm on the NCM811 particle surface. The fast Fourier transform (FFT) pattern obtained from the NCM811 region (lower‐right inset of Figure 2c) exhibited a distinct diffraction spot corresponding to the (003) plane of NCM811, suggesting that the layered structure of NCM811 was preserved after the LZO protective layer coating process. Figure 2d shows a high‐angle annular dark‐field scanning transmission electron microscopy (HAADF‐STEM) image (inset) along with the corresponding energy‐dispersive X‐ray spectroscopy (EDS) mapping results. The EDS mapping of Zr revealed a continuous and uniform distribution of Zr signals along the surface of the NCM811 particles, indicating that the LZO protective layer was conformally and uniformly deposited on the particle surfaces. This result is also observed in Figure S3 (Supporting Information). Figure 2e shows a comparison of the X‐ray diffraction (XRD) patterns of NCM811 with and without various LZO protective layers. The distinct diffraction peaks at 18.6° and 44.4°, corresponding to the (003) and (104) planes, respectively, barely shift or broaden upon LZO protective layer coating, indicating that the crystal structure of NCM811 was maintained. This result is consistent with the TEM results in Figure 2c and confirms that the ALD process for depositing the LZO protective layer hardly induces structural degradation in NCM811.

**Figure 3:**
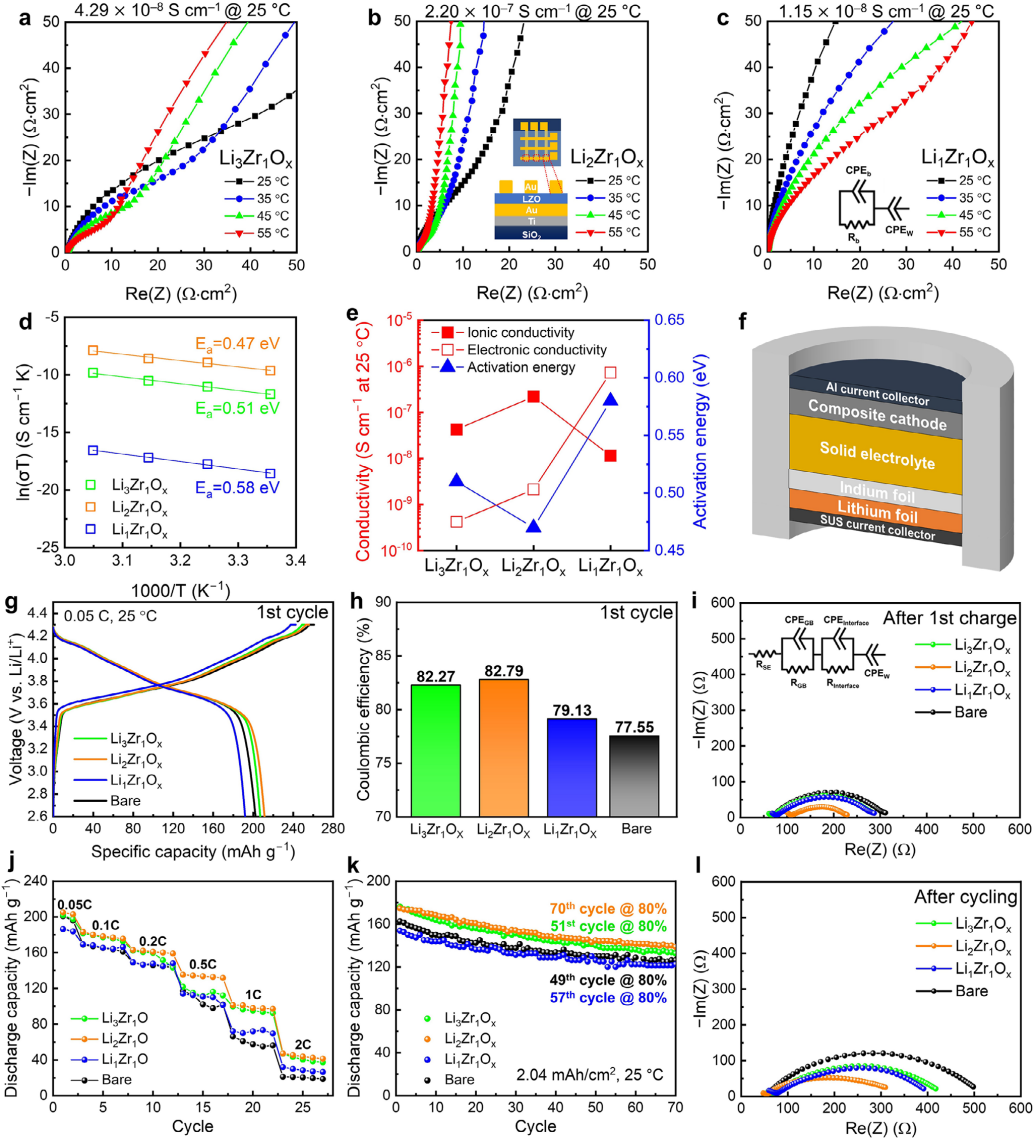
Nyquist plots of a) Li_3_Zr_1_O*_x_*, b) Li_2_Zr_1_O*_x_*, and c) Li_1_Zr_1_O*_x_* films. d) Arrhenius plots of ionic conductivity of various LZO films. e) Summary of ionic conductivity, electronic conductivity, and activation energy for various LZO films. f) Schematic of the all‐solid‐state cell (torque cell) fabricated for electrochemical estimation. Electrochemical performances of the cells with and without various LZO protective layers: g) charge–discharge profiles and h) CE at the first cycle, j) rate capability, and k) cycling performance. Nyquist plots for evaluating the impedance after i) first charge and l) cycling. All cells were operated in a controlled chamber maintained at 25 °C. Rate capability and cycling tests were conducted independently to ensure reliable evaluation.

To evaluate the ionic and electronic conductivities of LZO films with various compositions, a crossbar array device^[^ ^37^ ^]^ was fabricated on an Au‐patterned substrate (inset of **Figure** **3**b) and analyzed using electrochemical impedance spectroscopy (EIS). Figure 3a–c shows the Nyquist plots obtained for various LZO films at temperatures ranging from 25 to 55 °C. In the high‐frequency region, semicircles corresponding to the bulk resistivity of the LZO film are observed. An incomplete semicircle with an endpoint detached from the *x*‐axis is a common artifact when using ion‐blocking electrodes. This behavior, arising from the nonideal capacitive response owing to contact issues between the Au electrode and LZO film, was accommodated by replacing the ideal capacitor with a constant phase element (CPE) in the equivalent circuit model (inset of Figure 3c).^[^ ^38^, ^39^ ^]^ The ionic conductivity of LZO film depended on the film composition but barely increased monotonically with Li concentration. Li_2_Zr_1_O*_x_* film exhibited the highest ionic conductivity (2.20 × 10^−7^ S cm^−1^ at 25 °C), compared with Li_1_Zr_1_O*_x_* (1.15 × 10^−8^ S cm^−1^) and Li_3_Zr_1_O*_x_* films (4.29 × 10^−8^ S cm^−1^).

Figure 3d presents Arrhenius‐type plots of ionic conductivity for various films, derived from the EIS data in Figure 3a–c. The temperature dependence of the ionic conductivity is described by the following equation:

(1) $$\sigma T=\sigma_{0}\;\exp\left(-E_{a}/kT\right)$$

where *σ* is the ionic conductivity, *T* is the absolute temperature (K), *E_a_* is the activation energy, *k* is the Boltzmann constant, and *σ_0_* is the pre‐exponential factor. By converting this equation into its logarithmic form and plotting ln(*σT*) versus 1/*T*, *E_a_* was extracted from the linear slope. *E_a_* represents the energy barrier that Li ions must overcome during transport through the protective layer, and lower *E_a_* values correspond to lower energy barriers for ion movement. Notably, Li_2_Zr_1_O*_x_* film exhibited the lowest *E_a_* (0.47 eV). In contrast, both Li_3_Zr_1_O*_x_* (0.51 eV) and Li_1_Zr_1_O*_x_* (0.58 eV) films exhibited higher *E_a_*, further confirming the inverse relationship between *E_a_* and ionic conductivity. This trend supports the reliability of conductivity. The behavior can be attributed to a trade‐off between the increased concentration of mobile Li^+^ and the disruption of conduction pathways caused by excessive Li.^[^ ^40^, ^41^, ^42^ ^]^ A similar compositional trend was previously reported in Li*_x_* TaO_y_ films, where the highest conductivity was observed near the optimal composition (*x* ≈ 0.98). Beyond this ratio, the conductivity decreased owing to the formation of excess nonbridging oxygen and Li‐based byproducts such as Li_2_CO_3_, hindering the ion transport.^[^ ^23^ ^]^

Electronic conductivity measurements of various LZO films showed that Li_1_Zr_1_O*_x_* film exhibited the highest electronic conductivity (7.26 × 10^−7^ S cm^−1^), which decreased with increasing Li concentration in the film (Figure S4, Supporting Information). This behavior is attributed to the increased formation of the electrically insulating Li_2_CO_3_ phase under Li‐rich conditions, which hinders charge transport.^[^ ^23^ ^]^ Figure 3e summarizes the dependence of ionic conductivity, electronic conductivity, and activation energy on the film composition. Although higher ionic conductivity and lower activation energy generally facilitate Li^+^ transport and reduce the overpotential, the role of electronic conductivity is governed by a trade‐off between ensuring sufficient electrical insulation to prevent side reactions at the CAM/SE interface and maintaining adequate electron percolation paths. Collectively, these results demonstrate that careful compositional tuning of the protective layer enables tailored conductivity characteristics, thereby guiding the identification of the optimal LZO film composition for performance enhancement.

To examine the effect of the composition of LZO protective layers on the electrochemical performance, torque cell‐based ASSBs were fabricated using NCM811 coated with 10 nm‐thick LZO protective layers of varying compositions (Figure 3f). Figure 3g,h show the first‐cycle charge–discharge profiles and the corresponding CE values, respectively. The initial performance varied depending on the ionic and electronic conductivities of the protective layers, which influenced interfacial transport and stability. Specifically, ionic conductivity governs Li^+^ transport kinetics and overpotential,^[^ ^25^ ^]^ while electronic conductivity contributes to the suppression of parasitic reactions by limiting electron transfer.^[^ ^43^ ^]^ Li_2_Zr_1_O*_x_* film exhibited the highest ionic conductivity (2.20 × 10^−7^ S cm^−1^ at 25 °C) and a low electronic conductivity (≈10^−9^ S cm^−1^), enabling efficient Li^+^ transport and suppression of parasitic interfacial reactions. As a result, the cell fabricated with Li_2_Zr_1_O*_x_* protective layer (Li_2_Zr_1_O*_x_* cell) achieved the highest discharge capacity and CE of 211.3 mAh g^−1^ and 82.79%, respectively. Li_3_Zr_1_O*_x_* film had the lowest electronic conductivity (≈10^−10^ S cm^−1^) but suffered from limited ionic conductivity (4.29 × 10^−8^ S cm^−1^). Accordingly, the cell fabricated with Li_3_Zr_1_O_x_ protective layer (Li_3_Zr_1_O*_x_* cell) exhibited a slightly reduced discharge capacity and CE of 207.1 mAh g^−1^ and 82.27%, respectively. Li_1_Zr_1_O*_x_* film exhibited both the lowest ionic conductivity (1.15 × 10^−8^ S cm^−1^) and the highest electronic conductivity (≈10^−7^ S cm^−1^), which caused increased transport resistance and sustained side reactions. Consequently, the cell fabricated with Li_1_Zr_1_O*_x_* protective layer (Li_1_Zr_1_O*_x_* cell) showed the lowest discharge capacity and CE of 191.9 mAh g^−1^ and 79.13%, respectively. The cell fabricated without a protective layer (bare cell) showed degraded performance, with a discharge capacity of 202.3 mAh g^−1^ and a CE of 77.55%, attributable to overpotential increases and irreversible capacity loss caused by an uncontrolled cathode–electrolyte interphase (CEI).^[^ ^6^ ^]^ Figure 3i presents the Nyquist plots of the various cells after the first charge (0.05 C to 4.3 V vs Li/Li^+^). The diameter of the semicircles reflects the interfacial resistance between the cathode and the SE.^[^ ^44^, ^45^, ^46^ ^]^ The Li_2_Zr_1_O*_x_* cell exhibited the lowest AC impedance (≈126 Ω), while the Li_3_Zr_1_O*_x_* and Li_1_Zr_1_O*_x_* cells showed values of ≈200 Ω, and the bare cell had the highest resistance (≈244 Ω). These results align with the trends observed in the discharge capacity and CE (Figure 3g,h), emphasizing that differences in conductivity are key drivers of the initial cell performances.

Figure 3j compares the rate capabilities of various cells with and without LZO protective layers of different compositions. The discharge capacity varied significantly depending on the protective layer's composition, and this difference became more pronounced as the C‐rate increased. Notably, at 1C, the Li_2_Zr_1_O*_x_* cell delivered a discharge capacity of 101.08 mAh g^−1^, ≈8.6% and 30.7% higher than the Li_3_Zr_1_O*_x_* (92.38 mAh g^−1^) and Li_1_Zr_1_O*_x_* (70.00 mAh g^−1^) cells, respectively. Compared to the discharge capacity of the bare cell (55.13 mAh g^−1^), the improvement reached ≈45.5%. Across all C‐rates, the Li_2_Zr_1_O*_x_* cell consistently maintained the highest discharge capacity, followed by the Li_3_Zr_1_O*_x_* and Li_1_Zr_1_O*_x_* cells, which are primarily attributed to variations in the ionic conductivity. At high C‐rates, where sluggish Li^+^ mobility becomes a limiting factor, the higher ionic conductivity of the protective layer helps minimize the interfacial resistance, thereby preserving the cell capacity.^[^ ^28^ ^]^

The cell lifespan is determined by multiple interacting factors, including the interfacial Li^+^ resistance, electronic conductivity of the protective layer, and structural or mechanical degradation of the CAM during repeated cycling. In particular, when the protective layer has high electronic conductivity, persistent redox reactions occur at the CAM–SE interface, leading to irreversible capacity loss. As a byproduct of these reactions, a CEI layer forms containing ionically resistive and electronically conductive species, which increases polarization and accelerates capacity fading.^[^ ^22^, ^26^, ^47^, ^48^ ^]^ Figure 3k compares the cycling performance of cells fabricated with and without various LZO protective layers over 70 cycles. The Li_2_Zr_1_O*_x_* cell demonstrated the best performance, delivering a capacity of 139.7 mAh g^−1^ with 80% retention after 70 cycles. This correlates with the Li_2_Zr_1_O*_x_* film's highest ionic conductivity (2.20 × 10^−7^ S cm^−1^ at 25 °C) and lowest electronic conductivity (≈10^−9^ S cm^−1^), which together minimized both transport resistance and parasitic reactions. In contrast, the Li_3_Zr_1_O*_x_* cell achieved a slightly lower capacity of 133.8 mAh g^−1^ at the 70th cycle and showed 80% retention after 51 cycles. Although its initial CE (82.27%) was comparable to that of the Li_2_Zr_1_O*_x_* cell (82.79%), the relatively low ionic conductivity (4.29 × 10^−8^ S cm^−1^) of the Li_3_Zr_1_O*_x_* film led to elevated interfacial resistance. While the low electronic conductivity (≈10^−10^ S cm^−1^) may have helped suppress side reactions, the limited Li^+^ transport ultimately constrained the long‐term cycling stability. In contrast, the Li_1_Zr_1_O*_x_* film exhibited the lowest ionic conductivity (1.15 × 10^−8^ S cm^−1^) and the highest electronic conductivity (≈10^−7^ S cm^−1^), which induced both sluggish Li^+^ transport and persistent side reactions. As a result, the Li_1_Zr_1_O*_x_* cell exhibited the poorest cycling performance with a capacity of 121.3 mAh g^−1^ at the 70th cycle and reached 80% retention after only 57 cycles. The bare cell showed severe degradation (125.0 mAh g^−1^ at the 70th cycle, 49 cycles at 80%) owing to uncontrolled CEI formation during cycling.

Figure 3l shows the Nyquist plots of the various cells measured after 70 cycles. The Li_2_Zr_1_O*_x_* cell maintained the lowest interfacial impedance (≈280 Ω), followed by the Li_1_Zr_1_O*_x_* (≈324 Ω) and Li_3_Zr_1_O*_x_* (≈358 Ω) cells, and the bare cell (≈459 Ω). Similarly, differential capacity (d*Q*/d*V*) plots (Figure S5, Supporting Information) and corresponding polarization values (Figure S6, Supporting Information) showed that the Li_3_Zr_1_O*_x_* and Li_1_Zr_1_O*_x_* cells exhibited values ≈7% and ≈5% higher, respectively, than those of the Li_2_Zr_1_O*_x_* cell, while the bare cell showed a 44% increase. These results demonstrate that the protective layer's composition directly influences the ionic and electronic transport properties, which collectively govern the long‐term cycling performance of the cell.

**Figure 4:**
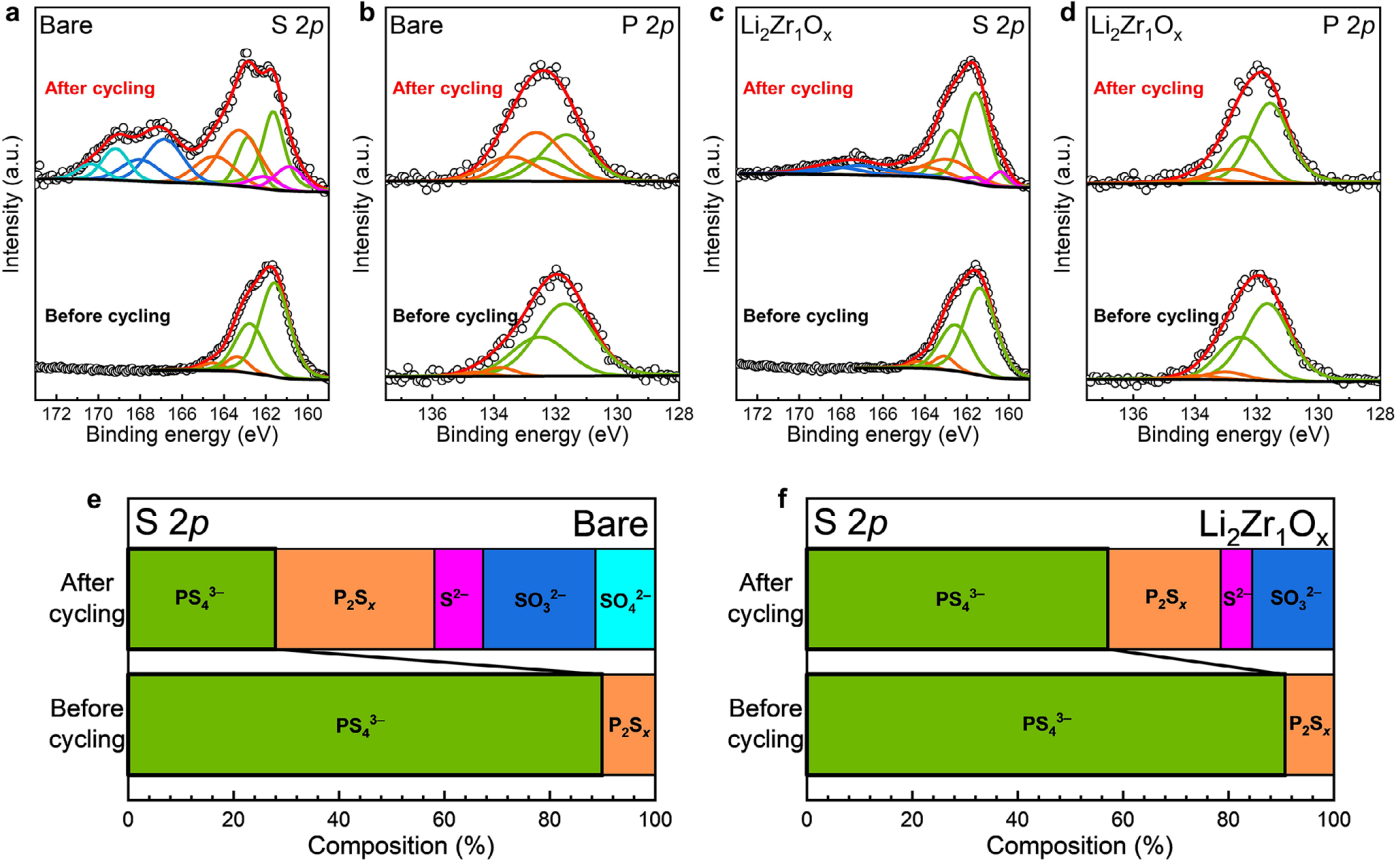
XPS analysis of bare and Li_2_Zr_1_O*_x_* cells before and after cycling. a) S 2*p* and b) P 2*p* core‐level spectra of the bare cell. c) S 2*p* and d) P 2*p* core‐level spectra of the Li_2_Zr_1_O*_x_* cell. Compositional analyses derived from the S 2*p* core‐level spectra for the e) bare and f) Li_2_Zr_1_O*_x_* cells.

**Figure** **4**a–d present the S 2*p* and P 2*p* core‐level spectra obtained by XPS for the bare and Li_2_Zr_1_O*_x_* cells before and after cycling, and the corresponding compositional analyses are shown in Figure 4e,f. For the bare cell (Figure 4a,e), the S 2*p* core‐level spectra before cycling exhibited a PS_4_^3–^ peak at the BE of 161.5 eV originating from Li_6_PS_5_Cl (LPSCl) SE (90.01%), together with a P_2_S*_x_* peak at the BE of 163.3 eV (9.90%). This peak is likely induced by direct contact between NCM or conducting agent (CA) and LPSCl. After cycling, the PS_4_^3^^−^ fraction decreased to 28.02%, while decomposition products of LPSCl increased. These include P_2_S*_x_* at the BE of 163.5 eV (30.08%), SO_3_^2–^ at the BE of 166.8 eV (21.31%), SO_4_^2–^ at the BE of 169.2 eV (11.35%), and S^2–^ at the BE of 160.8 eV (9.23%). The S^2^^−^ peak can be attributed to Li_2_S and NiS*_x_*. As a result, the total fraction of decomposition products of LPSCl increased to 71.97% after cycling. The P 2*p* core‐level spectra (Figure 4b) showed a consistent trend. The PS_4_^3–^ peak at the BE of 131.6 eV decreased after cycling, whereas P_2_S*_x_* species at the BE of 133–134 eV increased. These decomposition products are ionically resistive and electronically conductive species that increase interfacial resistance while not readily suppressing further oxidation of LPSCl, thereby explaining the deterioration of cell performance.^[^ ^22^, ^26^, ^47^, ^48^ ^]^ For the Li_2_Zr_1_O*_x_* cell (Figure 4c,f), the S 2*p* core‐level spectra before cycling showed PS_4_^3–^ at the BE of 161.5 eV (90.81%), together with a small P_2_S*_x_* contribution at the BE of 163.5 eV (9.19%) attributed to direct contact with CA. After cycling, the total fraction of LPSCl decomposition products, including P_2_S*_x_*, SO_3_^2−^, SO_4_^2−^ and S^2−^, decreased significantly to 42.81%, compared with 71.97% in the bare cell. The P 2*p* core‐level spectra (Figure 4d) also revealed only a slight increase in P_2_S*_x_* at the BE of 133–134 eV after cycling, much smaller than that observed in the bare cell (Figure 4b). Taken together, these results provide clear evidence that the Li_2_Zr_1_O*_x_* protective layer, with low electronic conductivity on the order of 10^−9^ S cm^−1^ and high ionic conductivity of 2.20 × 10^−7^ S cm^−1^, effectively suppresses oxidative decomposition of LPSCl during cycling. Furthermore, the comparison between the two extreme cases of bare and Li_2_Zr_1_O*_x_* cells directly demonstrates that the conductivity of the protective layer governs CEI formation, consisting of interfacial decomposition products at the CAM–SE interface.

## 3 Conclusion

This study provides direct experimental evidence that the composition of ALD‐grown LZO protective layers affects the ionic and electronic conductivities, which in turn dictate the cell performance. Unlike conventional H_2_O‐based processes, the O_3_‐based ALD process used in this study enabled self‐limiting growth with a linear correlation between subcycle ratio and film composition, and reduced surface Li_2_CO_3_ phase formation by ≈50%. Compositional tuning resulted in LZO films with ionic conductivities in the range of 1.15 × 10^−8^–2.20 × 10^−7^ S cm^−1^ and electronic conductivities spanning three orders of magnitude. These differences in conductivity caused measurable variations in the electrochemical performance of the cells fabricated with the various LZO protective layers. The initial discharge capacity varied from 191.9 to 211.3 mAh g^−1^, and the CE ranged from 79.13% to 82.79%, representing composition‐dependent deviations of up to 10% and 4.6%, respectively. After 70 cycles, discharge capacity ranged from 121.3 to 139.7 mAh g^−1^, and the cycle life (to 80% retention) varied from 51 to 70 cycles, corresponding to a maximum difference of 37% depending on composition. Compared to the cell without a protective layer, the cell with Li_2_Zr_1_O*_x_* protective layer exhibited a 6.7% higher initial capacity and a 43% longer cycle life until 80% capacity retention. XPS analysis after cycling further confirmed that the Li_2_Zr_1_O*_x_* protective layer suppressed parasitic interfacial reactions at the NCM–LPSCl interfaces. Collectively, these findings not only reveal the critical role of the protective layer composition in controlling the conductivity and interfacial stability, but also provide a quantitative basis for rational coating design in next‐generation ASSBs.

## 4 Experimental Section

### Film Preparation

The Si substrate (4‐inch, *p*‐type, <100> CZ, 1–10 Ω cm, 500 ± 25 µm) was cleaned using a dilute 10% hydrofluoric acid (HF) solution and subsequently rinsed. LZO films were deposited on the cleaned Si substrates at 200 °C using a 4‐inch traveling‐wave‐type thermal ALD reactor. LiO*t*Bu and TEMAZ were used as the Li and Zr precursors, respectively. H_2_O and O_3_ (180 g m^−3^) served as oxygen sources. LiO*t*Bu, TEMAZ, and deionized (DI) water were maintained at 140, 55, and 10 °C, respectively. High‐purity Ar gas (99.999%) was used as both the carrier and purge gas.

### Powder ALD

ALD of LZO protective layer on NCM811 powder was conducted using a rotary‐type powder ALD system (ALPES Co., Ltd.). A cylindrical reactor containing NCM811 powder was placed in a preheated reactor at 200 °C and rotated for ≈1 h to ensure uniform heating of the powder. LZO layers with varying compositions were deposited using the supercycle method, as shown in Figure 1a. During the exposure of the precursor and oxygen source, the reactor was rotated for the mechanical dispersion of the powder to enhance adsorption onto the powder surface. After the exposure, a reactor isolation step was employed for a specific duration to ensure the deposition of a conformal thin film.

### Film Characterization

The thicknesses of the LZO films were measured using spectroscopic ellipsometry (MG‐1000, Nanoview Co.). The chemical states of the LZO films were analyzed by XPS (K‐Alpha, Thermo Fisher Scientific Co.), with the binding energy calibrated using the C─C bonding peak (285 eV) in the C 1*s* spectra. For CEI analysis before and after cycling, XPS (Nexsa, Thermo Fisher Scientific Co.) measurements were performed. To avoid exposure to air and moisture, the samples were prepared in an Ar‐filled glovebox and transferred to the analysis chamber using a sealed transfer holder. The Zr layer density was measured using EDXRF (ARL QUANT'X, Thermo Fisher Scientific Co.), and the compositions of the LZO films were determined using ICP‐MS (iCAP RQ, Thermo Fisher Scientific Co.). The elemental depth profile of the LZO film was obtained using Auger electron spectroscopy (AES; PHI 700 Xi, Physical Electronics). The thicknesses and morphologies of the LZO films were examined using a 200 kV field‐emission TEM (FE‐TEM; JEM‐2100F, JEOL). The TEM specimens were prepared by focused ion beam etching (Quanta 3D FEG, FEI).

### Electrochemical Impedance Spectroscopy Analysis

The electrochemical properties of the LZO films were evaluated in terms of their ionic conductivities using a cross‐bar array structure. First, the SiO_2_ substrate was cleaned by ultrasonic immersion in isopropyl alcohol and DI water for 10 min each. Subsequently, a 50 nm‐thick Ti adhesion layer and a 100 nm‐thick Au bottom electrode were deposited through a shadow mask using an e‐beam evaporator (iNFOVION), followed by ALD deposition of the LZO thin film. Finally, a 100 nm‐thick Au top electrode was deposited using a shadow mask. EIS was conducted using a potentiostat (ZIVE SP1, WonA Tech Co.) in the frequency range of 1 MHz–1 Hz.

### Electrical Measurement

The electronic properties of the LZO thin films were evaluated for electronic conductivity using a metal–insulator–metal structure. A 50 nm‐thick Ti adhesion layer and a 100 nm‐thick Au bottom electrode were deposited on a low‐doped *p*‐type SiO_2_ substrate using an e‐beam evaporator (iNFOVION), followed by the deposition of a 10 nm ALD LZO film. Finally, an Au top electrode was deposited through a shadow mask patterned with 200 µm‐diameter dots. The average current was measured under a 2 V DC bias applied for 30 min using a semiconductor parameter analyzer (4200A‐SCS, Keithley).

### Preparation of Composite Cathode

To fabricate the composite cathode, NCM811 (Rov Co.), LPSCl (Nanocamp Inc.), and Super P (Imerys) were used as the CAM, SE, and CA, respectively. The SE exhibited an ionic conductivity of at least 1 mS cm^−1^, as shown in Figure S7 (Supporting Information), and the same SE was used to fabricate both the composite cathode and ASSB.

For preparation, the materials were mixed at a CAM:SE:CA weight ratio of 80:19:1 using a vortex mixer under an Ar atmosphere with 2 mm‐diameter ZrO_2_ balls. LZO‐coated NCM811 powder was processed using the same ratio and procedure.

### Torque Cell Fabrication and Electrochemical Test

First, 100 mg of LPSCl was placed inside a cylindrical insulator with a 10 mm‐diameter hole, and a hydraulic press was used to apply 25 MPa pressure to form an SE pellet. Subsequently, 10 mg of the composite cathode (≈2.04 mAh cm^−2^ from 200 mAh g_CAM_^−1^) was uniformly spread on the SE pellet surface, followed by the placement of a 10 mm‐diameter Al current collector. Pelletization was performed at a pressure of 35 MPa.

On the opposite side, a 9 mm‐diameter In foil, 4 mm‐diameter Li foil, and 10 mm‐diameter stainless steel (SUS) foil were sequentially stacked. The assembly was then sealed within a SUS housing, and a preload screw was tightened to 11 N m using a torque wrench to maintain a constant pressure.

All electrochemical tests were conducted using a battery test system (CT‐4008Q, Neware) in a chamber maintained at 25 °C. The rate capability test was performed at 0.05, 0.1, 0.2, 0.5, 1, and 2C in a voltage range of 2.0–3.7 V versus In/Li (2.6–4.3 V vs Li/Li⁺). Specifically, the 0.05C step was conducted for two cycles using the constant current/constant voltage (CC/CV) mode for charging and the constant current (CC) mode for discharging, while the remaining steps were performed in CC mode for five cycles each. The cycling test was conducted in the voltage range of 2.0–3.7 V versus In/Li (2.6–4.3 V vs Li/Li⁺). Initially, the first two cycles were performed at 0.05C using the CC/CV mode for charging and the CC mode for discharging. Afterward, charge/discharge cycling was performed at 0.1C/0.2C in CC mode for 70 cycles. The charge–discharge curves of the rate capability and cycling tests are presented in Figures S8 and S9 (Supporting Information), respectively.

## Conflict of Interest

The authors declare no conflict of interest.

## Supporting information



Supporting Information

## Data Availability

The data that support the findings of this study are available from the corresponding author upon reasonable request.
