# Supplementary material for: Composition‐Controlled Cathode Protective Layer via Powder‐Atomic Layer Deposition for All‐Solid‐State Batteries
Source: Adv Sci (Weinh). 2025 Oct 14;13(4):e14583. doi: 10.1002/advs.202514583 (PMC12822380; doi:10.1002/advs.202514583)
Supplement: Supplementary file 1 — Supporting Information [file ADVS-13-e14583-s001.docx]

Supporting Information

**Composition-Controlled Cathode Protective Layer via Powder-Atomic Layer Deposition for All-Solid-State Batteries**

*Kyu Moon Kwon, Dae Ho Kim, Ha Yeon Kwon, Joungwon Park, Kyoung Hwan Kim, Hwi-Yeol Park, Hyo Rang Kang*, and Tae Joo Park**

K. M. Kwon, D. H. Kim, H. Y. Kwon, H. R. Kang, T. J. Park
Department of Materials Science and Chemical Engineering, Hanyang University, Ansan 15588, Korea

E-mail: tjp@hanyang.ac.kr

J. Park, K. H. Kim, H.-Y. Park
Battery Material TU, Samsung Advanced Institute of Technology (SAIT), Samsung Electronics Co., Ltd., Suwon 16678, Korea

H. R. Kang
Nanocamp Inc., Chungju 27315, Korea
E-mail: hrkang@nanocamp.kr

T. J. Park
ALPES Co., Ltd., Ansan 15588, Korea


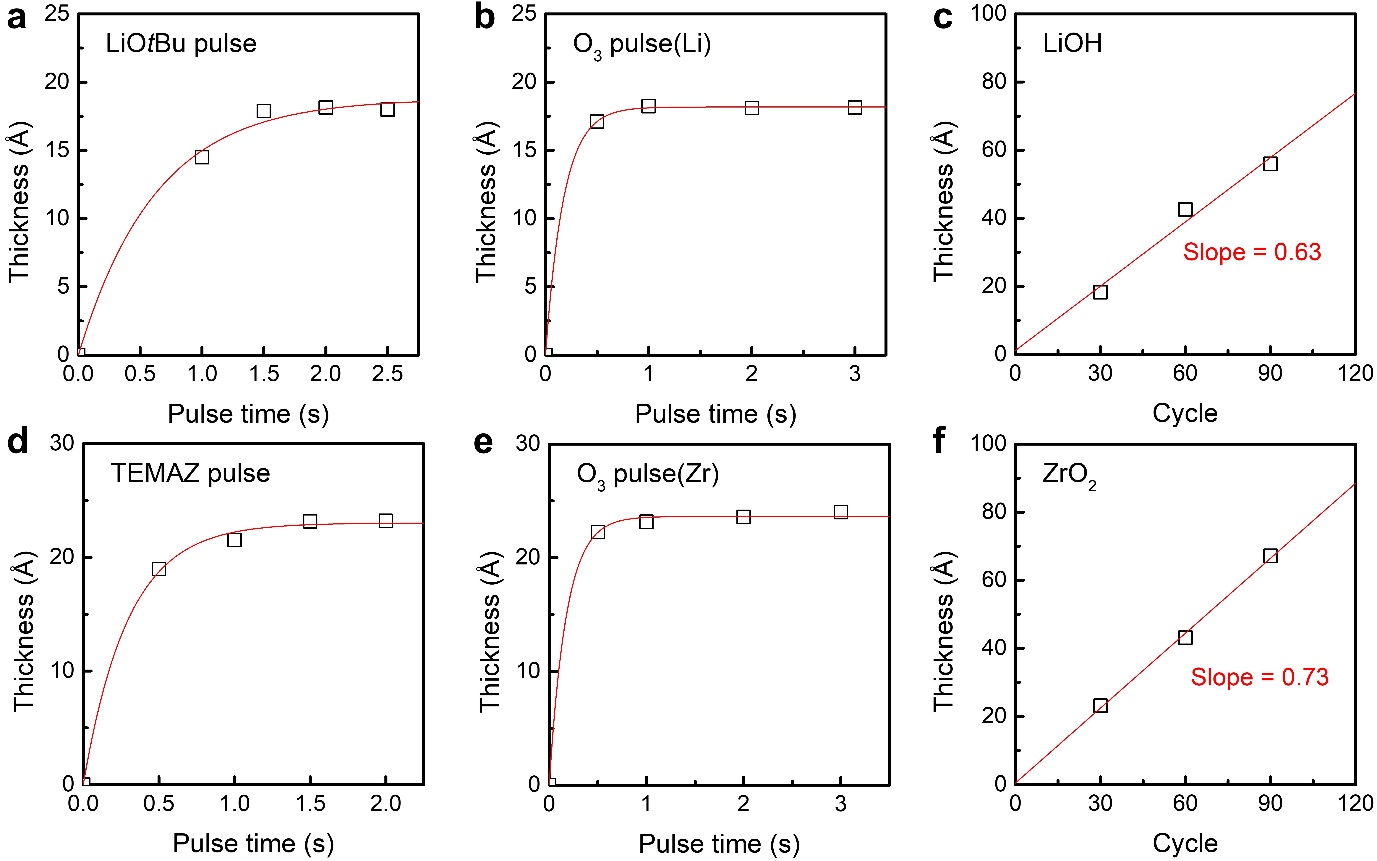


**Figure S1.** Pulse time saturation curves for (a, b) Li and (d, e) Zr subcycle on the Si substrate, clearly exhibiting self-limiting behavior characteristic of ALD processes. Film thickness as a function of cycle number for (c) LiOH and (f) ZrO_2_, where the slope obtained from linear fitting represents the growth rate(GR) of each corresponding film (GR = 0.63 Å cycle^–1^ for LiOH; GR = 0.73 Å cycle^–1^ for ZrO_2_).


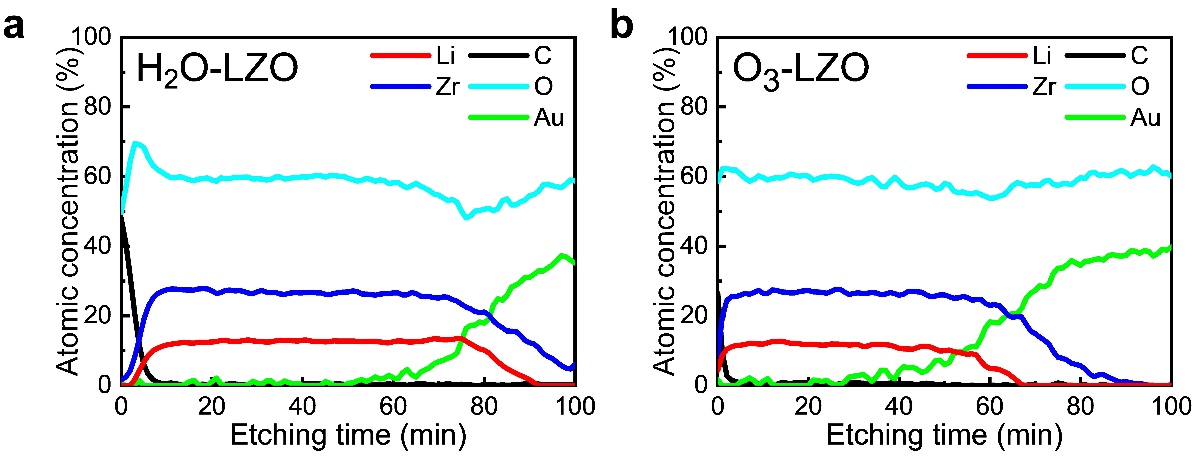


**Figure S2.** AES depth profiles of (a) H_2_O-LZO and (b) O_3_-LZO. Compared to H_2_O-LZO, O_3_-LZO exhibits a weaker carbon impurity signal near the surface (black line), while both samples show negligible atomic concentration of carbon in the bulk region, supporting the suitability of the O_3_-based process for LMO deposition.


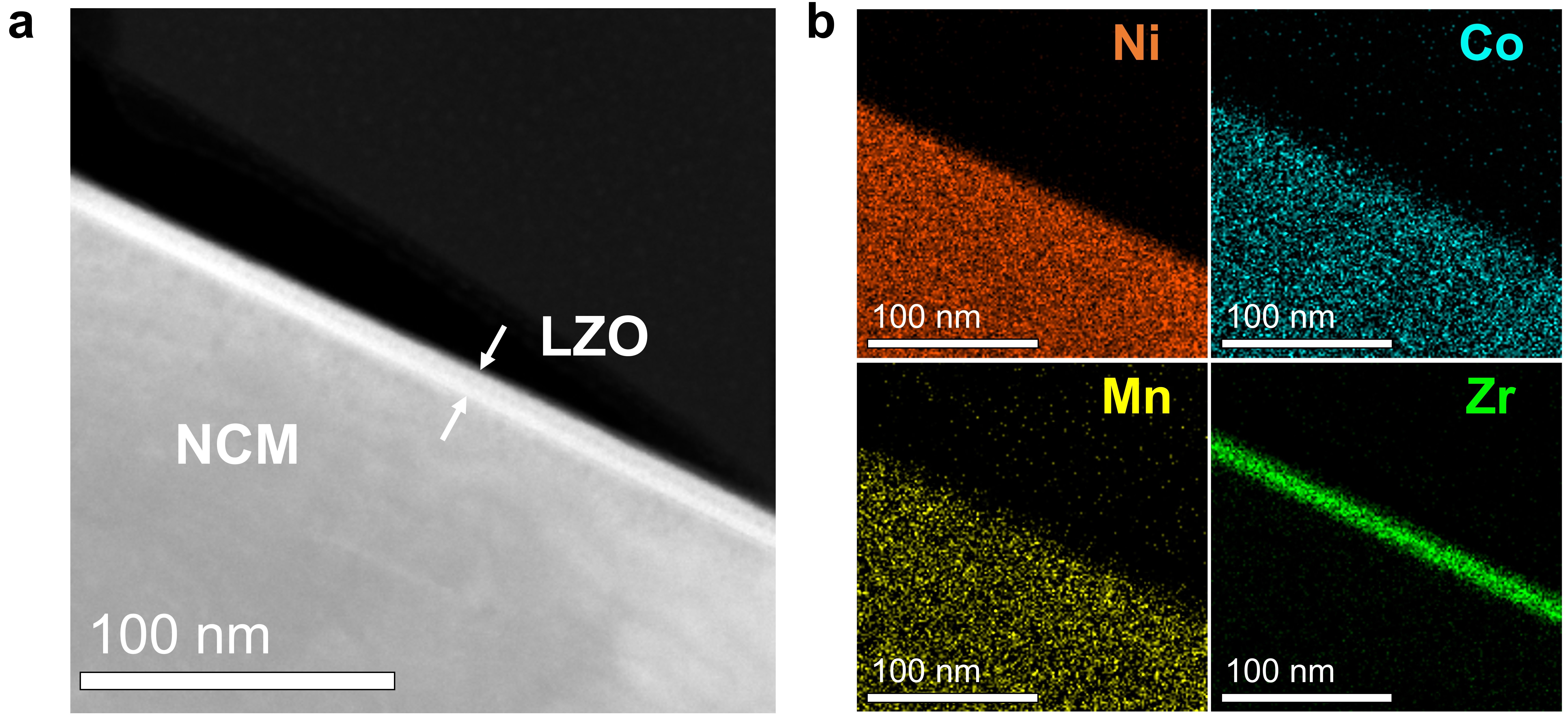


**Figure S3.** (a) HAADF-STEM image and (b) EDS elemental maps of NCM811 particle with the LZO protective layer. The uniformly distributed Zr signal along the NCM811 surface indicates the conformality of the LZO protective layer.


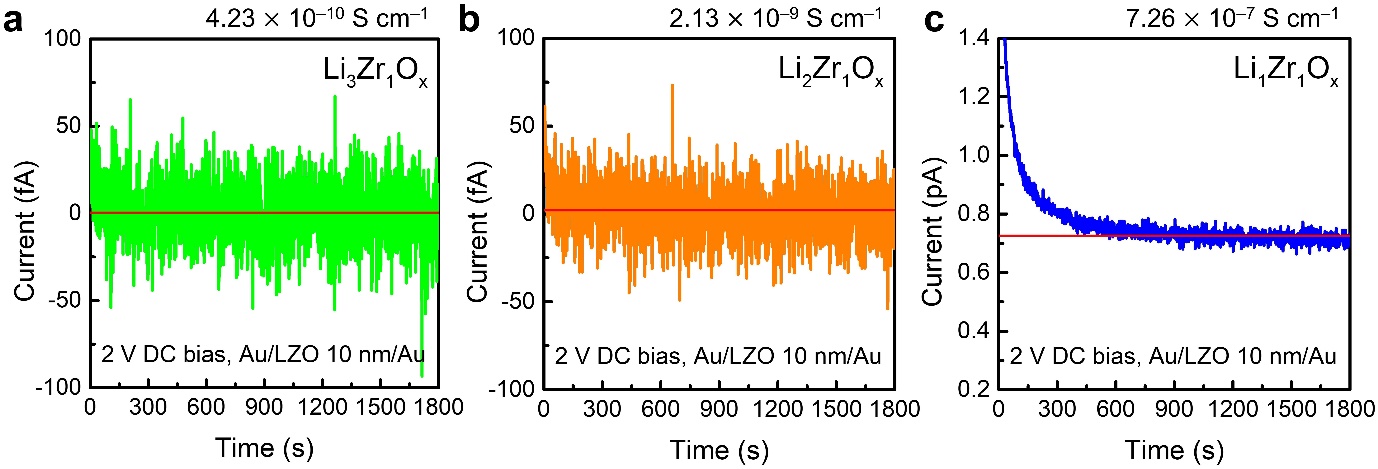


**Figure S4.** Current–time profiles of (a) Li_3_Zr_1_O*_x_*, (b) Li_2_Zr_1_O*_x_* and (c) Li_1_Zr_1_O*_x_* films measured under a constant DC bias of 2 V. The red lines represent the average current values over time, obtained through linear fitting.


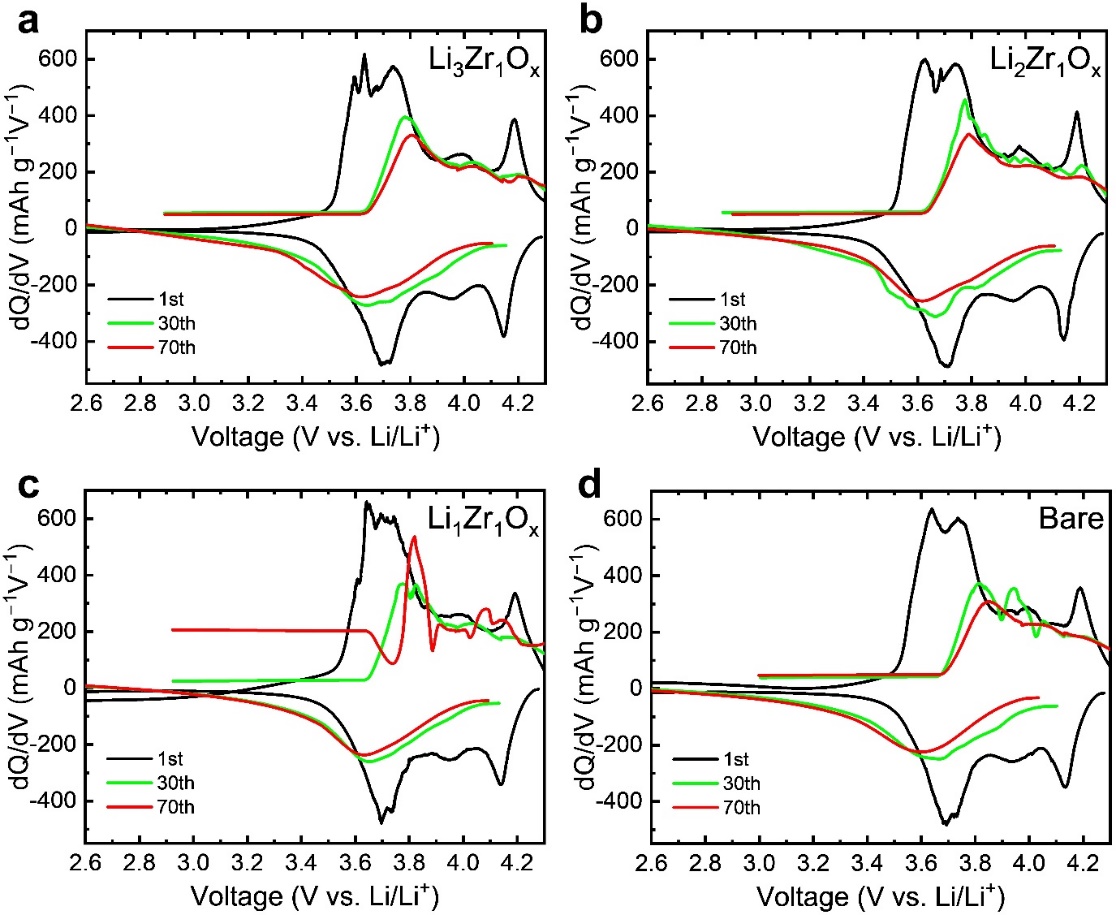


**Figure S5.** d*Q*/d*V* plots derived from the charge–discharge profiles at the 1st, 30th, and 70th cycles for the (a) Li_3_Zr_1_O*_x_*, (b) Li_2_Zr_1_O*_x_*, (c) Li_1_Zr_1_O*_x_* and (d) bare cell.


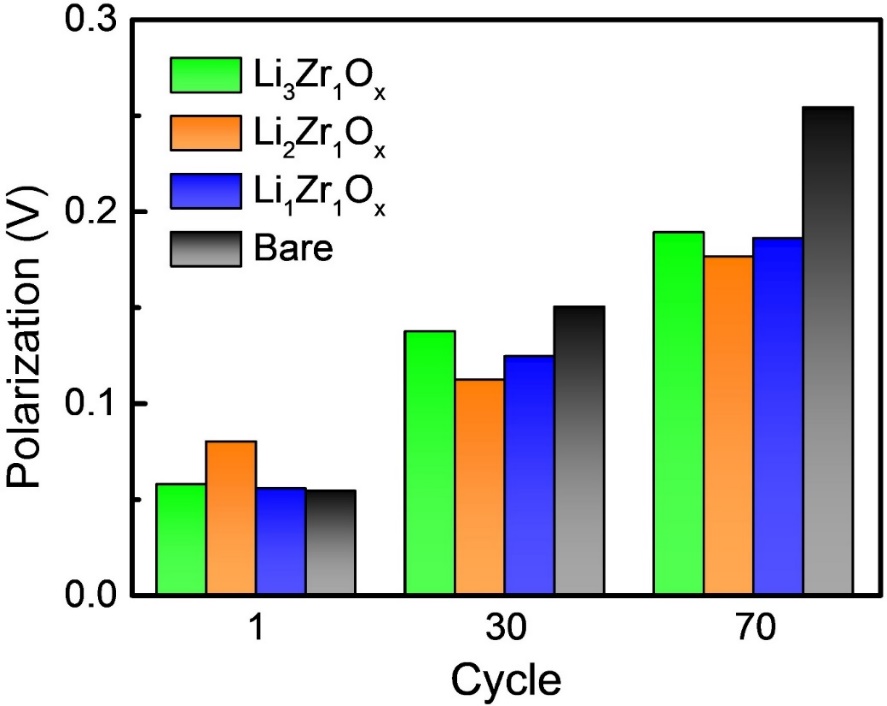


**Figure S6.** Polarization estimated from d*Q*/d*V* plots for the Li_3_Zr_1_O*_x_*, Li_2_Zr_1_O*_x_*, Li_1_Zr_1_O*_x_* and bare cell. Polarization was quantified as the voltage difference between the dominant anodic and cathodic peaks in d*Q*/d*V* plots.


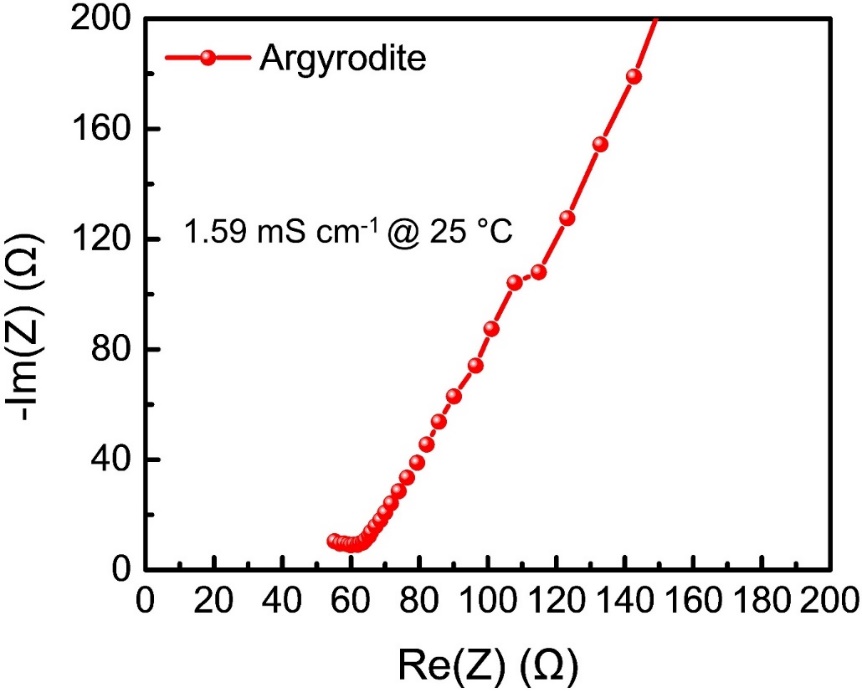


**Figure S7.** Nyquist plot of LPSCl SE used in this study for both the composite cathode and ASSB fabrication, exhibiting a high ionic conductivity exceeding 1 mS cm^–1^.


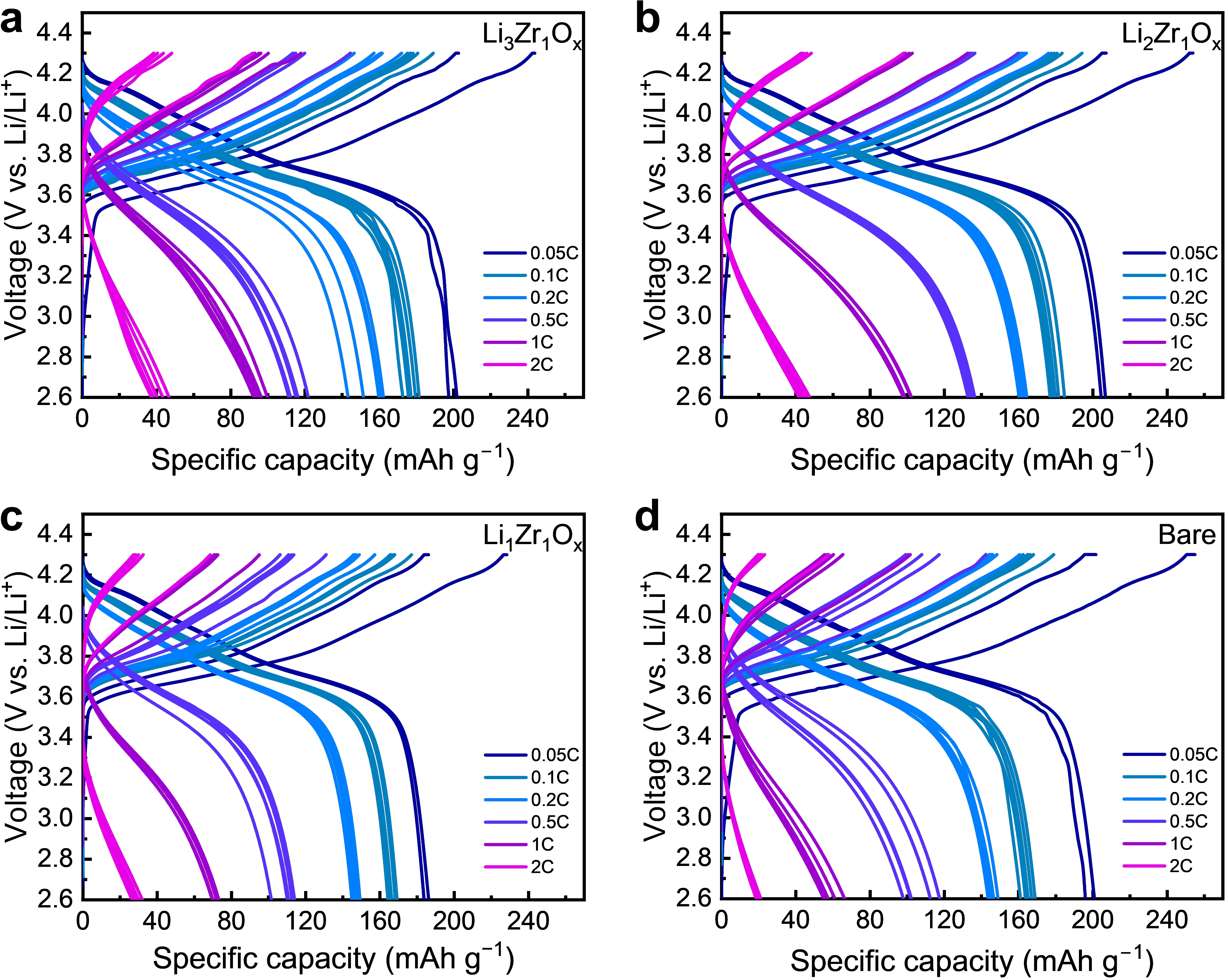


**Figure S8.** Charge–discharge curves in the voltage range of 2.0–3.7 V versus In/Li (2.6–4.3 V versus Li/Li^+^) at different C-rates (0.05C, 0.1C, 0.2C, 0.5C, 1C, 2C) for the (a) Li_3_Zr_1_O*_x_*, (b) Li_2_Zr_1_O*_x_*, (c) Li_1_Zr_1_O*_x_* and (d) bare cell.


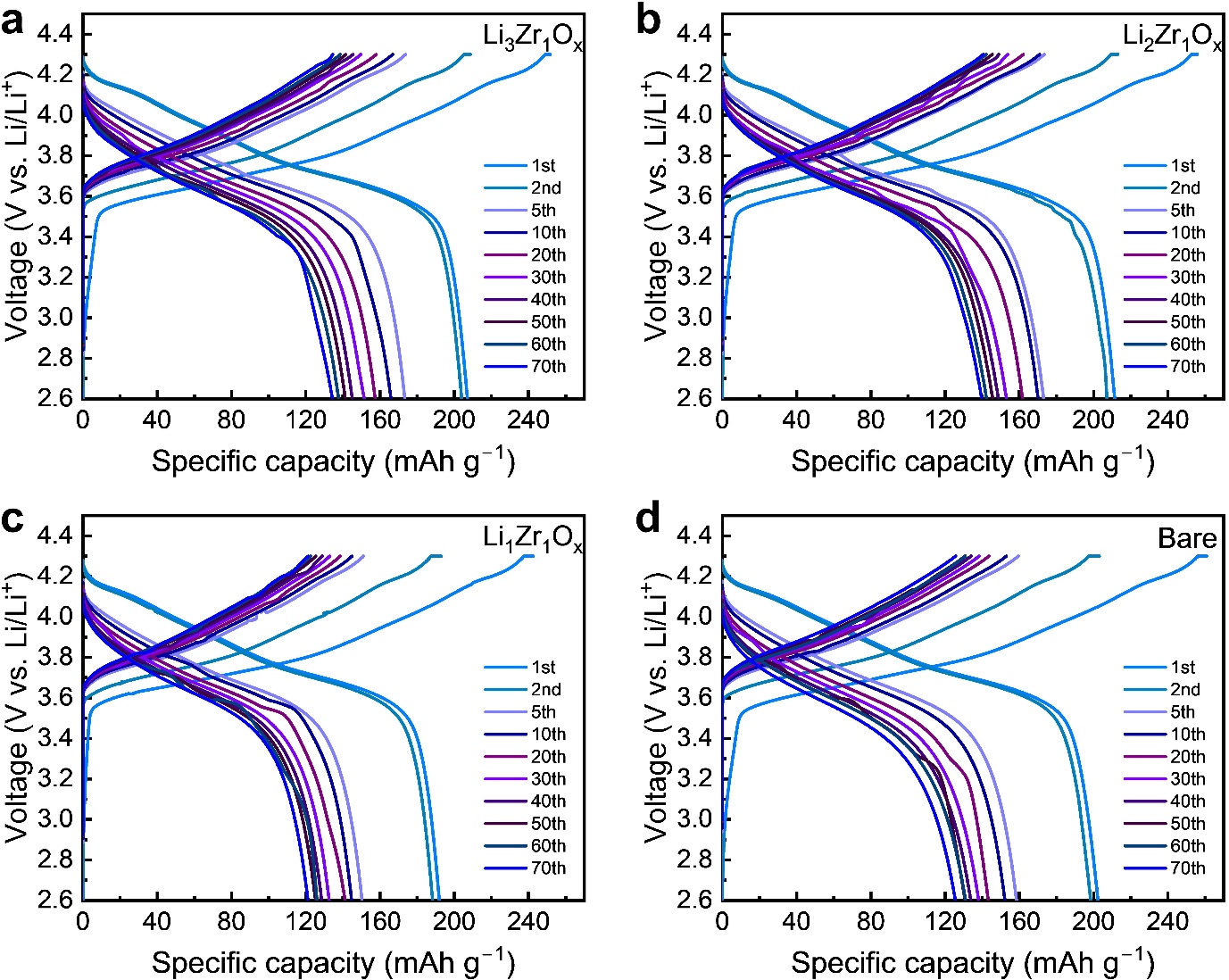


**Figure S9.** Charge–discharge curves in the voltage range of 2.0–3.7 V versus In/Li (2.6–4.3 V versus Li/Li^+^) during cycling (1st to 70th) for the (a) Li_3_Zr_1_O*_x_*, (b) Li_2_Zr_1_O*_x_*, (c) Li_1_Zr_1_O*_x_* and (d) bare cell.
